# The Population Problem: The Importance of Maternal Mortality as a Factor

**Published:** 1915-12-04

**Authors:** 


### THE POPULATION PROBLEM.

### THE IMPORTANCE OF MATERNAL MORTALITY AS A FACTOR.

FOR some years the number of intelligent citizens of both sexes who take a serious interest in the future of our race has been steadily increasing; and many sides of the problem of rearing a plentiful and a healthy "next generation" have received keen attention. By voluntary effort, mainly, distinct progress has been made in the direction of tackling the appalling infant mortality which lately disgraced us and is by no means yet sufficiently reduced. The falling birth-rate has attracted the attention of others, a few of whom are not discomposed by it, while the majority regard it as a catastrophe. There is officially on record the fact that if the birth-rate in England and Wales had been the same in 1914 as it was in 1876, there would have been born in the former year 467,837 infants more than were in fact actually born. The war and its wastage will most certainly force all these problems into greater prominence; and we may hope that the nation, and especially its elected representatives, may be stirred to much greater and better directed efforts in the future in obtaining control over the avoidable causes of human wastage that have been draining our population in the past.

In a supplement to the Report of the Medical Officer of the Local Government Board for 1914-15, the very important question of maternal mortality in connection with child-bearing is considered by Dr. Newsholme; and the whole Report is full of valuable information and suggestions. Figures are apt to make dull reading, but handled as they are in this Report they enhance the interest and drive home the moral more forcibly than any other form of argument could do. In the four years 1911-14 the number of maternal deaths assigned to child-bearing and all its complications was just over 14,000, a rate of about 4 per 1,000 births. Incidentally it may be remarked that this is certainly an underestimate, though perhaps not by a great deal. This mortality rate of 4 per 1,000 can be roughly divided into two sections, that due to "puerperal fever," and that due to other complications of childbirth. Since 1895 the mortality from the former has been steadily and markedly declining, and is now smaller than that of the latter category, which has not declined to anything like the same extent. While it is satisfactory that "puerperal fever" is diminishing, it has also to be admitted that it is still far too frequent; and that, as it is almost a preventible disease, the toll it levies is still a stain upon our national conscience. Attention has more than once in the last few years been drawn in THE HOSPITAL to the unreliability of the returns of puerperal fever; and it is interesting to learn that the Registrar-General is taking steps to improve them, with some measure of success. This means that a larger proportion of the true number of deaths from puerperal sepsis is being registered, and perhaps accounts for the slight inferiority of the figures for 1914 to those of the three preceding years. Not only does the death of women in connection with childbirth deprive the nation of fertile mothers who might reasonably have been expected still further to enrich the population had they lived; it tells also in another way by depriving their children of maternal care, and so conduces—to a minute extent, it is true—to an increased child mortality.

### A SLUR UPON WALES.

For reasons into which the Report does not inquire closely, Wales contrasts very unfavourably with England in regard to maternal mortality. Out of fifteen counties in which puerperal fever notifications are actually fewer than deaths from this cause, six are Welsh counties; and Dr. Newsholme is clearly satisfied that the notification of this "disease" is largely evaded in the Principality. Nor is it only "puerperal fever" that destroys a higher proportion of Welsh than of English mothers. The statistics show that the other causes of mortality are even more excessive in Wales; the total mortality rate from all causes is 5.75 per 1,000 births in Welsh, as against 3.97 in English counties. The explanation of this excess may be partly the inaccessibility of skilled help in remote and mountainous districts; and in this connection it is of interest that Westmorland shows the highest rate of any English county, while Cumberland, Devon, and Cornwall are also high up on the list. Another group of English counties which are prominent on the list are the industrialised shires of Lancashire, Yorkshire (West Riding), Durham, and Northumberland. Cheshire stands third to Westmorland and Lancashire, the reason for which evil prominence the Report cannot assign.

### MORTALITY IN INDUSTRIAL TOWNS.

The figures tell heavily against industrial towns also: Dewsbury, Rochdale, Burnley, Blackburn, Bury, Halifax, Merthyr Tydvil, Huddersfield, and Oldham have dreadful figures, all in excess of 6 per 1,000. The London boroughs come very well out of the test; they occupy, with few exceptions, very lowly positions on the list, and it is calculated that if the mortality of the rest of England and Wales had been the same as that of the Metropolis, 3,306 mothers would have been saved during the four years. After a careful summary of the available evidence, Dr. Newsholme declines to draw any general conclusion as to the geographical distribution of mortality from child-bearing; and suggests that special local factors, not merely topographical, are concerned in determining the amount of mortality from child-bearing.

What, then, are the factors which govern maternal mortality? For it is obvious that to improve matters we must find out both the favourable and the unfavourable determinants. Briefly, and omitting the statistics by which the results are buttressed, the majority of the deaths are caused by "puerperal fever," hæmorrhage, and convulsions. Most cases of these conditions, though not all, are well within the range of preventive medicine, including in this the early treatment of illness. The experience of the lying-in hospitals shows better results than does the average experience of the whole country; and when it is remembered that an unduly high proportion of complicated and dangerous cases are dealt with by these hospitals, the comparison becomes all the more striking.

The Value of Skilled Assistance.

It is idle to inquire whether the medical profession or the trained nurse-midwife can show the better statistics; for there are wide differences in the social and industrial conditions in which they work such as would certainly vitiate any comparison; nor do the statistics reveal any reliable ground for making one. Not even in respect of the trained midwife and the Gamp are the statistics definite enough to form a safe basis for inference, chiefly because there is insufficient information from each community as to the quality of the midwifery and the degree to which skilled help can be obtained when it is required. "On general grounds, however, there can be no reasonable doubt that the quality and availability of skilled assistance before, during, and after childbirth are probably the most important factors in determining the remarkable and serious differences in respect of mortality from child-bearing." The comparative experience of England, and of the other parts of the United Kingdom, and the contrasts between various counties and boroughs, confirm this conclusion.

The textile towns, it is pointed out, stand pre-eminent in the toll of life exacted from mothers in child-bearing; and it is probable that unsatisfactory midwifery bears an important share in producing this result. There must be, apart from this, a close relation between factory employment of women and excessive puerperal mortality, though the data do not permit Dr. Newsholme to be dogmatic. He finds considerable evidence that the use of abortifacients has an unfavourable influence, but the use of these is not confined to textile districts. Factory employment operates in two ways: pregnancy diminishes the industrial competence of the married woman, and so dangerous steps are sometimes taken to put a premature end to it; and industrial occupation during the later months of gestation increases the liability to dangerous complications before and during parturition. Sanitary defects have apparently little bearing upon puerperal mortality.

The Remedies.

In Dr. Newsholme's main Report to the Local Government Board, issued not so very long ago, he appeared to be in favour of the notification of pregnancy as one of the measures for facilitating pre-natal care (The Hospital, October 30, 1915, p. 93). This proposal he now rejects, on the strength of arguments which do not in themselves seem cogent enough to account for so complete a change of opinion. Ante-natal work by means of consultations and clinics, together with home visiting, is mentioned with approval; as a corollary may be added the provision of skilled assistance at maternity centres. The notification of births is regarded as essential for the proper collection of information; and it is advanced that the notification of "puerperal fever" should be made more effective. We quite agree that the present slipshod system of notifying "puerperal fever" is unsatisfactory and useless: the question, which has been on a previous occasion discussed in these columns, is whether it might not be as well to abolish it altogether, since it is perfectly certain that nothing approaching complete notification will ever be in practice obtained.

Finally, the Report contains some rather indefinite contributions to the admittedly difficult problem of the training and supply of midwives. A review of the position as to the distribution of midwives shows that in some areas the supply of midwives is inadequate to the public needs. Further, the inspection of midwives is done to a very varying extent in different counties and county boroughs. The statistics as to sending by midwives for medical help do not at present justify any general inference. There is also a most valuable appendix upon the economic aspects of midwifery as a calling for women, by Dr. Janet Lane-Claypon. This appendix is too full of facts and figures for summary here, but can be recommended in the original to all who are interested in the supply of instructed midwives for rural or urban communities.

